# The complete mitochondrial genome of red-fronted parrot (*Poicephalus gulielmi*) revealed a new gene rearrangement within the order *Psittaciformes*

**DOI:** 10.1080/23802359.2017.1407691

**Published:** 2017-11-25

**Authors:** Adam Dawid Urantówka, Aleksandra Kroczak, Paweł Mackiewicz

**Affiliations:** a Department of Genetics, Wroclaw University of Environmental and Life Sciences, Wroclaw, Poland;; b Department of Genomics, Faculty of Biotechnology, Wrocław University, Wrocław, Poland

**Keywords:** Duplication, mitogenome, *Poicephalus gulielmi*, Psittaciformes, rearrangement

## Abstract

Vertebrate mitogenomes are thought to be selected for compactness. Therefore, the increasing number of avian mitogenomes comprising duplicated regions is surprising. Such regions were proposed for at least 26 parrot genera based on the length of PCR products. However, complete mitogenomes with the duplications were shown only for six genera. These duplications evolved probably from the ancestral tRNA^THR^/tRNA^PRO^/ND6/tRNA^GLU^/CR and were subjected to subsequent degeneration. Here, we report the mitogenome of *Poicephalus gulielmi* (the subfamily Psittacinae) with a unique duplication tRNA^THR^/pseudoND6/CR1/tRNA^PRO^/ND6/tRNA^GLU^/CR2. This region is different from all other identified regions and resembles mostly the arrangements in *Amazona* and *Pionus* from the subfamily Arinae.

A typical mitogenome of vertebrates includes only one control region and the same set of 37 genes coding for two ribosomal RNAs, 13 proteins, and 22 tRNAs (Lavrov 2007). The coherent gene content makes that mitogenomes are still regarded to be under selection for compactness. However, the growing number of fully sequenced avian mitochondrial genomes has revealed that rearrangements and partial duplications occur in different lineages.

Recent studies also provided evidences for the persistence of duplicated CRs in parrot mitochondria. So far, 26 parrot genera were found to have a duplicated CR based on differences in the length of mitogenome fragments obtained in PCR (Schirtzinger et al. 2012). However, complete mitogenomes with duplicated elements were sequenced only for six genera: *Amazona*, *Forpus*, *Melopsittacus*, *Pionus*, *Prioniturus*, and *Psittacus* (Urantowka et al. 2013, 2016, 2017a; Eberhard and Wright 2016; Urantówka and Mackiewicz 2016). All identified genome rearrangements preserve two control regions and seem to evolve from the ancestral tandem duplication of tRNA^THR^/tRNA^PRO^/ND6/tRNA^GLU^/CR fragment followed by degeneration and/or loss of some genes (Eberhard and Wright 2016).

So far, *Psittacus erithacus* is the only representative of the *Psittacinae* subfamily with the known complete mitogenome sequence. This subfamily comprises also another genus *Poicephalus*, which in contrast to monotypic *Psittacus*, is the most species-rich and widely distributed in Africa. *Poicephalus* is very morphologically diverse (Forshaw 2010) and several of its species (*fuscicollis*, *gulielmi*, *senegalus*, *flavifrons*, and *meyeri*) are further divided into subspecies (Gill and Donsker 2017). Therefore, this parrot offers an interesting possibility to study mechanisms of speciation and emergence of new lineages. The previously recognized *Poicephalus robustus* taxon was recently divided into *r. robustus* (Cape parrot) and two other subspecies for which a new species *Poicephalus fuscicollis* (brown-necked parrot) was established: *f. fuscicollis* and *f*. *suahelicus* (Gill and Donsker 2017, see also for Hockey et al. 2005; Coetzer et al. 2015 for such proposition). This separation is also indicated by our results (Figure 1). However, this classification was questioned by some taxonomists because of small number of markers used. Therefore, further analyses of complete mitochondrial genomes are necessary to resolve this taxonomic proposition because results for single individual markers can be biased and produce inconsistent phylogenies (Urantówka et al. 2017b).

**Figure 1.**
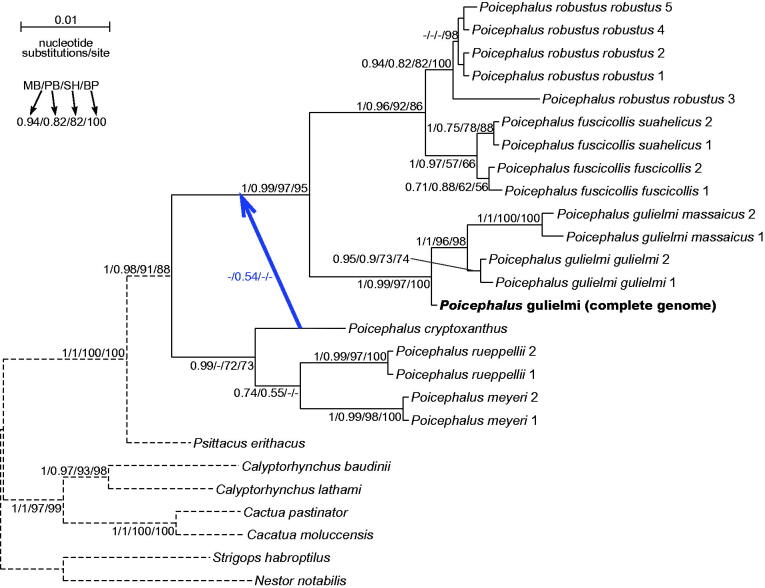
The phylogenetic tree obtained in MrBayes for the concatenated alignment of 16S rRNA and COI genes (1133 bp) indicating that the studied individual (bolded) belongs to *Poicephalus gulielmi*. Its sequences show the smallest genetic distance (0.49%) to *Poicephalus gulielmi gulielmi*. The individual is a male kept in culture in Poland (Kościan town) and naturally this species inhabits Central Africa. The blood sample, from which DNA was isolated, is available for other researches in the laboratory at the Department of Genetics in Wroclaw University of Environmental and Life Sciences under the number AUPMAK11. The blue arrow indicates an alternative position of clades in the PhyloBayes tree. The length of branches (the dashed lines) leading to outgroup sequences was shortened five times. The values at nodes, in the order shown, indicate posterior probabilities found in MrBayes (MB) and PhyloBayes (PB) as well as SH-aLRT (SH) and non-parametric bootstrap (BP) percentages calculated in IQ-TREE. The posterior probabilities <0.5 and the percentages <50% were omitted or indicated by a dash ‘–’. In the MrBayes (Ronquist et al. 2012) analysis, we assumed separate mixed substitution models for 16S rRNA and three codon positions in COI gene because PartitionFinder (Lanfear et al. 2012) proposed these four partitions as separate. We applied two independent runs, each using 4 Markov chains. Trees were sampled every 100 generations for 10,000,000 generations. After obtaining the convergence, trees from the last 5,429,000 generations were collected to compute the posterior consensus. In PhyloBayes (Lartillot and Philippe 2004), we applied CAT + GTR + Γ model and two independent Markov chains which were run for 50,000 generations with one tree sampled for each generation. The last 30,000 trees from each chain were collected to compute posterior consensus trees after reaching convergence. In the case of IQ-TREE (Nguyen et al. 2015), we used separate nucleotide substitutions models for two partitions as suggested by ModelFinder (Chernomor et al. 2016; Kalyaanamoorthy et al. 2017). In SH-aLRT bootstrap analysis, 10,000 replicates were assumed, and in non-parametric bootstrap, 1000 replicates were applied. The analysed sequences were downloaded from GenBank database under the accession numbers: AY309456, JF414239–42, KM611472/4, KP856835–52, KP856863–80. The tree demonstrates the significant separation of *Poicephalus robustus* and *fuscicollis*, which were formerly classified to one species. In comparison to result by Coetzer et al. (2015), the presented tree includes larger support values as well as groups together *P. rueppellii* and *P. meyeri*, and next adds to this clade *P. cryptoxanthus*. All these species distinguish from other studied *Poicephalus* species by smaller body length, 21 and 22 cm in comparison to 28 and 33 cm (Forshaw 2010). The grouping *P. rueppellii* and *P. meyeri* agrees also with their common morphological feature, i.e. yellow feathers on the leading edge of the wings.

Therefore, to enrich the set of molecular markers for future study of *Poicephalus* diversification, we obtained the sequence of mitogenome from *Poicephalus gulielmi* (accession number MF977813). Interestingly, the gene order found in the duplicated region tRNA^THR^/pseudoND6/CR1/tRNA^PRO^/ND6/tRNA^GLU^/CR2 is different from that identified by Eberhard and Wright (2016) for closely related *Psittacus erithacus*: tRNA^THR^/CR1/tRNA^PRO^/ND6/tRNA^GLU^/CR2. The gene order in *Poicephalus gulielmi* differs from any other previously identified and resembles mostly the arrangement characteristic of *Amazona* (Eberhard et al. 2001) and *Pionus* from the subfamily Arinae: tRNA^THR^/pseudoND6/psuedotRNA^GLU^/CR1/tRNA^PRO^/ND6/tRNA^GLU^/CR2.

The morphology of the analysed captive individual is typical of *gulielmi* species, which is undoubtedly confirmed in the phylogenetic analyses using 16S rRNA + COI alignment and including all available *Poicephalus* taxa (Figure 1). The specimen groups significantly with four other representatives of its species.
